# Building on the successes of patient-focused drug development: a call for new policies to maintain momentum

**DOI:** 10.3389/fmed.2024.1416123

**Published:** 2024-10-18

**Authors:** Allison Martin, Victoria DiBiaso, Mladen Bozic, Alexander T. May

**Affiliations:** ^1^Sanofi Genzyme, Framingham, MA, United States; ^2^CSL Behring, King of Prussia, PA, United States

**Keywords:** patient-focused drug development, patient experience data, Food and Drug Administration, PDUFA, regulatory decision making, benefit–risk, regulatory submissions

## Abstract

Since its commencement as part of the Food and Drug Administration’s (FDA) Prescription Drug User Fee Act (PDUFA) V in 2012, patient-focused drug development (PFDD) has become an integral part of the drug development paradigm. FDA encourages the development and use of Patient-Experience Data (PED) as it provides important information on the patients’ needs and perspectives and inform regulatory decision-making. While the FDA is required to fill out a table which includes a list of various types of Patient Experience Data (PED) and if such data was reviewed by FDA as part of a drug application, there is still a need to understand how FDA uses PED in its regulatory decision-making. This article examines whether new policies are needed to ensure the full potential of PFDD.

## 1 Introduction

Since its commencement as part of the Food and Drug Administration’s (FDA) Prescription Drug User Fee Act (PDUFA) V in 2012, patient-focused drug development (PFDD) has become an integral part of the drug development paradigm.

FDA defines PFDD as “a systematic approach to help ensure that patients’ experiences, perspectives, needs, and priorities are captured and meaningfully incorporated into drug development and evaluation” (1). An essential part of PFDD is patient experience data (PED) which includes data collected from patients intended to provide information about their experience with a disease or condition (2). These data can include information related to the burden of disease, symptoms, functioning and quality of life, experience with treatments, outcomes of importance, and preferences. Examples of PED could include, but are not limited to, patient preference surveys, natural history studies, Voice of the Patient Reports from PFDD meetings, or focus group interviews.

Since 2012, FDA has taken steps to incorporate PED into the regulatory decision-making process. This includes establishing the Center for Drug Evaluation and Research (CDER)‘s PFDD program which aims to bring the patient perspective to the forefront of drug development. Under this program, FDA has released a series of four methodological PFDD guidance documents, developed a PFDD Clinical Outcome Assessment (COA) Pilot Grant Program, and held PFDD meetings to solicit input from patients and patient advocates (1). FDA has also established the Patient Engagement Collaborative (PEC) which brings together FDA staff, patients, and patient advocacy groups to develop best practices for incorporating patient perspectives into the regulatory process (3).

Overall, FDA recognizes that PED can provide valuable insights into the safety and effectiveness of medical products and is committed to incorporating this information into its regulatory decision-making. However, work remains to ensure that generation of PED continues to gain momentum. Equally important is much needed clarity from FDA about how PED is being utilized during drug evaluation process.

## 2 The challenge

In an effort to help determine the extent of FDA’s utilization of PED, a 2016 law (21^st^ Century Cures Act, or “Cures Act”) directed the FDA to report on the use of PED in regulatory decision making (4). As part of the Cures Act, FDA is required to complete a PED table which includes a list of various types of PED and provide a statement explaining if PED or related information was submitted and reviewed by FDA as part of the application. In an assessment carried out by Eastern Research Group (ERG) on FDA’s uptake and application of the 2016 law (5), it was clear that there had been significant uptake of the use of the PED Table by FDA reviewers. However, a new regulatory gap emerged. What remains unclear is *how* FDA considered the submitted PED during the drug evaluation process. The ERG report found that while 68% of reviews referenced PED and 82% of those included a PED table, it was not clear whether the PED was considered valuable and usable data by FDA. The table also does not allow for any indication of how the PED data was applied or factored into regulatory decision-making. Additional findings from the report reflected wide variation in whether and how FDA uses PED in application approval decisions, inconsistencies in the inclusion of the PED table in review documents, the need for greater clarity and specificity in FDA expectations around PED, and FDA’s tendency to focus on endpoints that can be easily measured or of interest to clinicians, rather than those that reflect psychosocial, quality or life, or functional ability (5). The collective findings of the report suggest a central theme—the need for greater clarity around how FDA uses PED in regulatory reviews and decision-making.

Sponsors must consider various approaches to evidence generation during the early stages of product development, and all development programs strive to predict the right combination of data collection exercises to characterize anticipated benefit and risk. In addition to a lack of understanding around how FDA considers submitted PED, it also remains unclear how the Agency evaluates PED relative to other sources of data. While the industry generally understands that clinical data derived from two randomized controlled trials represent the “gold standard” of evidence generation, such studies are not always practical or feasible depending on the indication, especially for products intended to treat patients with rare diseases. Analyses of relationships between patient experience and clinical safety and effectiveness have indicated that positive associations between PED, safety, and effectiveness are consistent across disease areas, study designs, settings, populations groups, and outcome measures, while negative associations are rare (2.5%) (6). Such data supports the use of PED in clinical studies as it is directly related to measuring safety and effectiveness (6).

Increased transparency into the weight FDA review teams assign to different forms of PED (e.g., Patient Reported Outcomes (PRO), COAs, PFDD meeting outputs, patient preference study results, etc.) compared to each other as well as clinical and observational sources, would enable sponsors to make more informed regulatory strategy decisions about how and when to invest in PED generation. Acknowledging that FDA employs a single evidentiary standard, sponsors would welcome and have requested additional guidance clarifying whether the Agency’s thinking on the weighting of PED elements would change due to factors like small patient populations, high-risk patient populations (e.g., pediatric), intent to address unmet medical needs, or intent to address “life-threatening” conditions as defined in 21 CFR 312.81 (7).

Despite the still-evolving regulatory framework around utility of PED, a growing number of pharmaceutical companies have embraced the use of PED in product development and some industry members have even been prioritizing the patient voice before PFDD was an acronym. Sanofi, for example, began integrating the patient perspective over 10 years ago, and the company’s development programs are now 100% informed by individuals from the patient communities. Recently, in Sanofi’s Phase 3 study for Venglustat in Fabry Disease, PERIDOT (Patient ExpeRIence in Fabry Disease On VenglustaT), PED emphasized that pain in the extremities and abdomen were the most burdensome symptoms, which shaped a PRO to measure those symptoms and the benefit of the drug. This approach was endorsed by the FDA (8).

Additionally, work with patient advisors and other key healthcare stakeholders highlighted the impact that treatment related toxicity and tolerability can have a negative impact on quality of life, and potential treatment adherence. As a result of early exploration of patient experiences across multiple oncology indications, Sanofi-developed the Patient Qualitative Assessment of Treatment version 2 (PQATv2) PRO for use in early phase oncology studies. This novel fit-for-purpose PRO aims to generate data earlier in the development phase to improve the selection of later phase PROs and endpoints to reflect the PED most relevant to patients and their caregivers (9).

At CSL Behring, patient feedback has been employed to modify elements in number of studies, including study procedures, study visits, eligibility criteria, study duration, and endpoint selection, to improve patient access to much needed treatment options. The patient perspective is also used to inform new study capabilities, such as a patient concierge model, portal, and navigator program, to support recruitment and ensure that study populations reflect disease populations as closely as possible in alignment with FDA’s guidance on demonstrating clinically meaningful benefit. Patient advocacy groups have been at the forefront of the development of patient-experience data and have devoted time and funds to developing many varieties of data including Voice of the Patient Reports (10), Benefit/Risk studies (11), and rigorous data on what outcomes matter most (12). These work products provide enormous value and insight on the patient experience, preferences, and risk-tolerances to both industry and FDA. However, until there is more clarity on how FDA incorporates these into their regulatory decision-making, PED’s potential impact on Benefit/Risk assessment in support of regulatory submissions remains unclear.

With the final FDA PFDD Draft Guidance required by PDUFA VI now published, new clarity is provided on the acceptability of patient-focused COA-based endpoint considerations and evaluating the meaningfulness of treatment benefit (13). While this is a welcomed next step from FDA, more transparency is needed to illuminate how and where FDA uses PED, including non-COA PED. While the pharmaceutical/biotechnology industry appreciate the advice in the PFDD guidance series and applauds the Agency’s willingness to be a global leader among other health authorities, releasing the final PFDD Draft Guidance more than a year later than the due date stated in the PDUFA VI commitment letter suggests slower progress around PFDD implementation and acceptance than envisioned (14–16).

## 3 New policies and practices are needed

As pharmaceutical companies, advocacy organizations, and patients continue to invest resources into collecting and integrating data on the patient experience into medicines development, there is an increasing need to understand how PED submitted to the FDA is being used. To maintain the momentum and uptick in the use of PED in regulatory submissions and decision-making, additional policies are needed to clarify if and how such data is being utilized.

A legislative proposal introduced in the United States Congress in February 2023 called the BENEFIT (Better Empowerment Now to Enhance Framework and Improve Treatments) Act attempts to address this need by requiring FDA to consider relevant PED in the benefit/risk framework and make a public statement of how the data was considered (17). For patients and industry, this would be a welcomed improvement to the current PED reporting processes at FDA.

Until this regulatory adjustment is made, there will still be uncertainty in how FDA is using PED and the risk that industry may devote resources elsewhere. For Patient-Focused Drug Development to continue to grow and reach its full potential, policy changes are needed to ensure that stakeholders consistently and better understand how FDA is using this important information in its regulatory reviews and decision-making.

The upcoming eighth re-authorization of PDUFA provides the opportunity to include commitments to provide additional transparency and consistency around the utilization and interpretation of PED during drug evaluations. One specific solution that could address the issue of transparency and consistency would be updating the FDA’s PED Table to include a column that captures a short summary of the PED data and their impact on the regulatory assessment and decision-making. This column would provide a place for FDA to share information consistently and reliably on the application of the PED, how or where FDA considered the data in the context of the review, and any conclusions which impacted the regulatory decision. Additionally, FDA should employ a standardized approach of how and where PED appears in the review documents so that it is easily located.

Moreover, PDUFA VIII commitments should focus on supporting a better understanding of transparency, engagement opportunities, and reasonable flexibilities that encourage wider adoption of patient centricity during drug development to ensure innovators address the needs of the patients they ultimately serve. For example, FDA should develop an online repository of PED tables and lay summaries for easy access and understanding by all stakeholders, including those without technical training. This repository would also aid in the assessment of whether the new policies are having their intended impact. Lastly, PDUFA VIII could include an evaluation of the impact of such transparency, like what the BENEFIT Act would introduce, to determine whether further enhancements in the regulatory framework are needed.

While the primary way to ensure clarity and transparency on the regulatory utility of PED is through new policies requiring better reporting from FDA, there are steps industry could take to make it more clear to FDA how PED included in submissions are intended to be used. For example, when PED are included in an application, industry could include language specifically referencing what part of the review those data are intended to impact (e.g., Benefit–Risk considerations). Industry could also populate the PED Table and include the completed table in its submission documents, consistent with the FDA Electronic Common Technical Document (eCTD) Technical Conformance Guide (18). Such practices may assist reviewers in completing the requirement to report on the use of PED in informing regulatory decision-making.

## 4 Conclusion

FDA’s Patient-Focused Drug Development initiative has been critical to ensuring that the patient perspective is incorporated into product development and regulatory reviews. While the use of PED by industry sponsors has continued to increase since its inception, the question of how FDA is using PED submitted in applications to inform its regulatory decision-making remains. Transparency and consistency around how the FDA considers PED during the drug evaluation process is essential to the use and continued growth of PFDD and new policies, such as those introduced by the BENEFIT Act, are needed to ensure the utilization of PED continues.
